# Peripheral Amino Acid Levels in Schizophrenia and Antipsychotic Treatment

**DOI:** 10.4306/pi.2008.5.4.203

**Published:** 2008-12-31

**Authors:** Vincenzo De Luca, Emanuela Viggiano, Giovanni Messina, Alessandro Viggiano, Carol Borlido, Andrea Viggiano, Marcellino Monda

**Affiliations:** 1 Department of Psychiatry, University of Toronto, Centre for Addiction and Mental Health, Toronto, Ontario, Canada.; 2 Section of Human Physiology 'Filippo Bottazzi', Department of Experimental Medicine, Second University of Naples, Naples, Italy.; 3 Department of Study of Institutions and Territorial Systems, University of Naples "Parthenope", Naples, Italy.

**Keywords:** Amino acids, Schizophrenia, Antipsychotics, Neuroleptics

## Abstract

Abnormal levels of amino acids have been reported in patients with schizophrenia and have also been investigated as a biomarker to monitor antipsychotic treatment, however results have been inconsistent. The purpose of the present review is to summarize the evidence in the literature of whether amino acid levels can be a biomarker and predict the treatment outcome in schizophrenia. The current review does not support amino acid concentration as a useful biomarker for monitoring antipsychotic response in patients with schizophrenia, although there is evidence that high levels of serum homocysteine and glutamate might be considered as a trait marker for schizophrenia. This review has also highlighted a considerable dearth of studies, specifically of studies evaluating antipsychotic side-effects.

## Introduction

The synthesis of biogenic amines (dopamine, norepinephrine, serotonin and histamine) is related to the uptake of their amino acid precursors tyrosine, tryptophan and histidine.^1^ The levels of these substrates in the central nervous system (CNS) are influenced by the blood concentration of valine, leucine, isoleucine, and phenylalanine, which have affinity for the same carriers of tyrosine and tryptophan to cross the blood-brain barrier.^2^ Other amino acids such as glycine, serine, glutamic acid and aspartic acid function as neurotransmitters in the brain and are involved in neuronal development.^3^ A chemical imbalance of these neurotransmitters have been postulated in the pathophysiology of schizophrenia. Thus, changes in amino acid plasma concentrations might affect the susceptibility to psychotic disorders and influence their treatment outcome.

According to this hypothesis, many researchers have monitored peripheral amino acid concentrations in patients with schizophrenia during the antipsychotic treatment. However, the changes in the serum amino acid concentrations were not replicated consistently across studies, and different investigators concluded that further work is required to better understand the relationship between the level of circulating amino acids and schizophrenia as well as to clarify its possible role in both the physiopathology of psychotic disorders and the response to neuroleptics and atypical antipsychotics.

The aim of this review is to summarize the evidence for the correlation between the levels of amino acids present in the blood and vulnerability for schizophrenia. Furthermore we reviewed the articles where the amino acid levels have been investigated as antipsychotic treatment predictors.

Medline was used to select appropriate papers that investigated amino acids in schizophrenia and antipsychotic treatment by matching the word schizophrenia and antipsychotics with the names of twenty proteinogenic amino acids. In addition, the following terms were employed: taurine, ornitine, citrulline and homocysteine. The abstracts of all studies generated by this searches were read by the first author to select appropriate studies which met the following inclusion criteria: i) only original research and published journal articles were included in the review; ii) the research must include the serum or plasma measurement of at least one amino acid in a sample of schizophrenia or psychotic subjects; iii) the study must have been written in English.

## L-tyrosine

L-tyrosine is a non essential aromatic amino acid. Our group^4^ found higher concentration of tyrosine without any change in the tyrosine/large neutral amino acid ratio in the serum of schizophrenia patients treated with clozapine. This amino acid is the precursor of dopamine, norepinephrine and epinephrine; therefore the lower concentration of tyrosine could be a peripheral marker of the hyperdopaminergic condition hypothesized to explain psychosis.^5^

## L-tryptophan

L-tryptophan is an essential amino acid and in several studies, researchers found lower concentration of tryptophan and a reduced tryptophan/large neutral amino acid (amino acids that compete with tryptophan for the uptake) ratio in the serum of schizophrenia patients. For example, Rao and colleagues^6^ and Tortorella and colleagues^7^ found lower levels of tryptophan in schizophrenia that can result in lower neuronal uptake of tryptophan, which may in turn produce lower serotonin levels in the CNS of patients with schizophrenia.

L-tryptophan is the precursor of serotonin therefore the opposite variation of this amino acid and tyrosine in schizophrenia could be a trait-marker of the chemical imbalance of dopaminergic and serotonergic transmission postulated in schizophrenia.^5^ It is also interesting that during the clozapine treatment our group observed a significant increase of the tryptophan level in patients with schizophrenia, even though the level was still significantly lower than controls after 12 weeks of clozapine therapy. Similarly Alfredsson and colleagues^8^ found an increase of tryptophan correlated to the improvement during the early part of the treatment.

## L-serine

Among the conditionally essential amino acids, L-serine is the most studied in schizophrenia for its role as cotransmitter, regulating the N-methyl-D-aspartate (NMDA) glutamate receptors. Several studies have shown that there are high levels of serine in patients with schizophrenia as a trait marker rather than a state marker.^9^ Waziri^10^ reported that serine is increased in both the serum and the brain of individuals affected by schizophrenia and suggested a possible role of serine in the pathophysiology of schizophrenia. Macciardi and colleagues have replicated these results,^11^ whereas other groups have reported^6^,^12^,^13^ normal level of serine in the plasma.

In contrast, by the mean of high pressure liquid chromatography we reported^4^ that serine levels were lower in drug-free schizophrenics and no change were shown in serine levels during the chronic antipsychotic administration even though there was a significant improvement of psychotic symptoms.

It is possible that the inconsistency between this result and the results in other studies mentioned above is due to the clinical feature of the Italian sample that consisted in resistant schizophrenics according the Kane's criteria.^14^ In fact, the long-lasting treatment with typical antipsychotics in resistant subjects might induce a permanent decrease of serine levels in the blood, not sensitive to the washout or the switch to clozapine. It is also interesting that Waziri and colleagues^10^ reported a decreasing effect of neuroleptics on serine concentrations in subjects with psychosis. In addition, inconsistent evidence among studies may account for the different methods used such as the measurement technique, time of blood draw, dietary intake and genetic difference in the studied populations may account for the controversial evidence. Among these biasing factors, the diet could be the most important confounder. In fact L-threonine free diet reduced the concentration of glutamic and aspartic acids in the nucleus accumbens of rat.^15^

## L-glutamine

L-glutamine is a non-essential amino acid. Alfredsson and colleagues^16^ did not find any change in the serum glutamine level of acutely psychotic patients compared to healthy volunteers. However, later the same group showed that high level of glutamine seems to predict the failure of sulpiride treatment^8^ and a significant negative correlation was shown between glutamine level and clinical response to sulpiride.

## L-asparagine

L-asparagine is a non essential amino acids. The level of asparagine was found to be lower in subjects with schizophrenia (after wash-out) than in matched controls in our previous report.^4^ However, no correlation was found between L-asparagine level and clozapine response.^4^ One case report found extremely high level of asparagine in schizophrenia-like psychosis^17^; however the metabolic correction of the hyperasparaginemia did not improve psychotic symptoms.^17^ Consistently Rao et al.^6^ found higher levels of asparagine in drug-free patients with schizophrenia than in healthy controls.

## L-glutamate

Glutamate is a non essential amino acid but it is the most important excitatory amino acid in the CNS. Our group^4^ found that glutamate was elevated in our sample of 11 subjects with schizophrenia even though Alfredsson and colleagues^18^ were unable to show any difference between schizophrenics and healthy volunteers. Our finding is consistent with Macciardi and colleagues who detected higher concentrations of glutamate in the serum of schizophrenia patients.^11^ Tomiya^19^ was able to show elevated serum level of glutamate only in male subjects with schizophrenia but not in females.

Among studies that have suggested that different levels of glutamate can be useful predictor for antipsychotic treatment, Tortorella and colleagues^4^ found that clozapine lowered, but was unable to normalize, the glutamate level. In contrast, Evins and colleagues^20^ found that the chronic treatment with clozapine produces increment of the glutamate in the blood. Furthermore Alfredsson and colleagues^8^ found that glutamate was higher in non-responders to sulpiride and that during the treatment the level of glutamate increased consistently with the clinical improvement. More recently Goff and colleagues^21^ found that Olanzapine treatment increases serum glutamate levels in patients previously treated with neuroleptics; finally, Maeshima and colleagues^22^ found that increment of glutamate is associated with the remission stage after antipsychotic treatment. The conflicting outcomes can be a result of the length of the follow-up significantly longer in the Evins' study. Finally, it is possible that the lengthy storage of the samples can produce artefacts in the glutamate detection.^16^

The glutamatergic hypothesis of schizophrenia^7^ postulates that the brain level of glutamate is lower in the brain of schizophrenic subjects and that antipsychotics operate as enhancers of the glutamatergic neurotransmission. However glutamate is not an essential amino acid; in fact it is actively synthesized in the CNS^3^ therefore the peripheral level does not reflect the amount of glutamate in the brain. More recently this lack of consistency between central glutamate and plasma glutamate has been confirmed by Goff et al.^21^ and Shulman et al.^23^ who showed that the peripheral glutamate does not correlate with the medial prefrontal cortical glutamate measured using the high-field strength proton magnetic resonance spectroscopy. However other studies have described a positive correlation between the level of glutamate in the cerebrospinal fluid and the serum.^16^,^24^

## L-aspartate

Aspartate is a non-essential amino acid. We found that aspartate was elevated in our sample of resistant schizophrenics after antipsychotic wash-out.^4^ Conversely, schizophrenic subjects switched to clozapine had no change after 12 weeks of treatment. Therefore, according to our data, aspartate and glutamate might be differently affected by the antipsychotic treatment. However, Evins et al.^20^ found that clozapine treatment increased the basal aspartate serum level.

## Glycine

Glycine is a non-essential amino acid and a inhibitory neurotransmitter; it plays also a role in the regulation of NMDA receptors. Higher concentrations of glycine were detected in the serum of schizophrenia patients compared to controls in some studies.^11^,^25^ However, Carl and colleagues,^13^ did not report any significant difference between schizophrenics and controls in terms of glycine level. According to Carl and colleagues^13^ we were unable to find any relationship between glycine and schizophrenia or clozapine administration.^4^

## L-proline

Proline is considered a conditionally essential amino acid. Jaquet et al.^26^ showed that moderate hyperprolinemia is a risk factor for schizophrenia and the serum proline levels are controlled by the enzyme proline dehydrogenase (PRODH). Furthermore Raux and colleagues^27^ found that subjects with velo-cardio-facial syndrome (VCFS), a condition that is often associated with schizophrenia, showed high levels of proline. It has been reported that 50% of the patients with VCFS are hyperprolinemic,^28^ and that 12 to 30%^29^-^31^ of them have psychosis.

## L-alanine

Alanine levels in schizophrenia have been poorly investigated. We did not find association between L-alanine and schizophrenia but we observed a reduction of this amino acid during a 12 weeks clozapine treatment.

## L-isoleucine

L-isoleucine is one of the eight amino acids (phenyalalnine, valine, threonine, tryptophan, isoleucine, methionine, leucine, and lysine) that are considered essential for humans and it is also considered a large neutral amino acid competing with tryptophan and tyrosine for the transporter across the blood brain barrier. This amino acid is a branched chain amino acid and in our previous work^4^ we found that the serum level of isoleucine was higher than in schizophrenia subjects compared to healthy controls. Higher levels of isoleucine in schizophrenia were found by Bjerkensedt et al.,^32^ too. However there is no correlation between isoleucine level and antipsychotic treatment.

## L-phenylalanine

L-phenylalanine is an aromatic amino acid. We did not find any significant difference in the level of phenyalanine when we compared schizophrenia individuals to controls. Furthermore the phenylalanine did not correlate with antipsychotic treatments. However Wei et al.^33^ found an imbalance of the tyrosine-phenylalanine ratio in early onset patients with schizophrenia.

## L-leucine

We found no variation in basal serum L-leucine associated either with schizophrenia or antipsychotics. However Reveley et al.^34^ found significantly higher levels of leucine in the cerebrospinal fluid (CSF) of schizophrenic patients.

We found that valine is not associated with schizophrenia. In contrast, Bjerkenstedt^32^ found higher concentrations of valine in unmedicated schizophrenia subjects compared to healthy controls.

## L-histidine

In our previous work,^4^ we found that the serum levels of L-histidine was higher in schizophrenia subjects compared to healthy controls. Our results were in agreement with Carl and colleagues^13^ who found that hystidine and other basic amino acids tended toward higher level in schizophrenia.

## L-arginine

L-ariginine is part of the urea cycle amino acid group. We did not find any correlation between the concentration of arginine and schizophrenia status or response to clozapine. However Carl et al.^13^ found a trend for higher level of arginine and other basic amino acids in schizophrenia.

## L-lysine

Lysine is an essential amino acid. Bjerkenstedt^5^ found that lysine is higher in the plasma of drug-free schizophrenics.

## L-threonine

L-threonine levels in schizophrenia have been poorly investigated. In our previous work, we found that L-threonine is associated neither with schizophrenia nor with clozapine response.^4^

## L-methionine

Baseline Methionine was found to be lower in nonresponders patients under atypical antipsychotic treatment.^35^ Furthermore Bjerkenstedt and colleague^32^ found higher methionine plasma concentrations in unmedicated schizophrenics. Finally Fekkes at al.^36^ found that the ratio of taurine and serine-methionine product was a useful diagnostic tool for acute psychosis.

## L-cysteine

We did not quantify L-cysteine in our sample. However Rao et al.^6^ found that this sulphur amino acid concentration is lower in drug-free schizophrenics.

## Homocysteine

Homocysteine is a non proteinogenic sulphur amino acid. A recent meta-analysis including 812 cases and 2,113 controls showed that higher levels of this amino acid are associated with schizophrenia.^37^

## Ornithine

Ornithine is an urea cycle amino acid. Tomiya et al.^19^ found that ornithine serum level is positively correlated with the length of illness. Furthermore in a case report Perry et al.^17^ found that ornithine were irregularly high in fasting plasma of an acute psychotic subject.

## Citrulline

Citrulline is classified as an urea cycle amino acid and it was found higher in drug-free schizophrenics by Rao et al.^6^

## Taurine

Taurine is an inhibitory neurotransmitter and was found significantly higher in drug-free schizophrenic patients.^32^ Taurine level in schizophrenia has been investigated by few researchers.

## Conclusions

The majority of the published papers reviewed, have examined amino acid plasma levels in the context of the dopaminergic, serotonergic and glutamatergic hypothesis of schizophrenia.

We have reviewed papers where amino-acid levels in schizophrenia patients have been compared to matched controls and manuscripts where amino acid levels have been used as predictors of treatment response.

Few early studies have employed the Structured Clinical Interview for DSM-IV (SCID)^38^ for diagnosing schizophrenia. Different rating scales have been utilized for assessing antipsychotic response. Consequently this hinders comparison between studies.

Most of the studies including our work,^4^ have employed the high-performance liguid chromatographic (HPLC) method, however few studies have employed gas chromatography or ion exchange chromatography, underlying that there is not standard method for quantifying plasma amino acids.

No working definition except for proline levels has been given as cut-off for defining an abnormal level. Previous research has not highlighted amino acid level differences between male and female, although Tomiya^19^ found sex differences in glutamate plasma levels.

With few exceptions, most of the studies reported herein found high glutamate and homocysteine plasma levels associated with schizophrenia. However these two indicators were rarely employed simultaneously.

Finally, it seems that few investigators have focused on the levels of amino acids and antipsychotic side-effects. However since there is new evidence that atypical antipsychotics produce metabolic changes, the serum amino- acid pattern might be an interesting tool to monitor metabolic side effects.
